# Immunosenescence in vertebrates and invertebrates

**DOI:** 10.1186/1742-4933-10-12

**Published:** 2013-04-02

**Authors:** Ludmila Müller, Tamas Fülöp, Graham Pawelec

**Affiliations:** 1 Max-Planck Institute for Human Development, Berlin, Germany; 2 Research Center on Aging, University of Sherbrooke, Sherbrooke, QC, Canada; 3 Second Department of Internal Medicine, University of Tuebingen Medical School, Tuebingen, Germany

## Abstract

There is an established consensus that it is primarily the adaptive arm of immunity, and the T cell subset in particular, that is most susceptible to the deleterious changes with age known as “immunosenescence”. Can we garner any clues as to why this might be by considering comparative immunology and the evolutionary emergence of adaptive and innate immunity? The immune system is assumed to have evolved to protect the organism against pathogens, but the way in which this is accomplished is different in the innate-vs-adaptive arms, and it is unclear why the latter is necessary. Are there special characteristics of adaptive immunity which might make the system more susceptible to age-associated dysfunction? Given recent accumulating findings that actually there are age-associated changes to innate immunity and that these are broadly similar in vertebrates and invertebrates, we suggest here that it is the special property of memory in the adaptive immune system which results in the accumulation of cells with a restricted receptor repertoire, dependent on the immunological history of the individual’s exposures to pathogens over the lifetime, and which is commonly taken as a hallmark of “immunosenescence”. However, we further hypothesize that this immunological remodelling *per se* does not necessarily convey a disadvantage to the individual (ie. is not necessarily “senescence” if it is not deleterious). Indeed, under certain circumstances, or potentially even as a rule, this adaptation to the individual host environment may confer an actual survival advantage.

## Introduction

**Figure 1:**
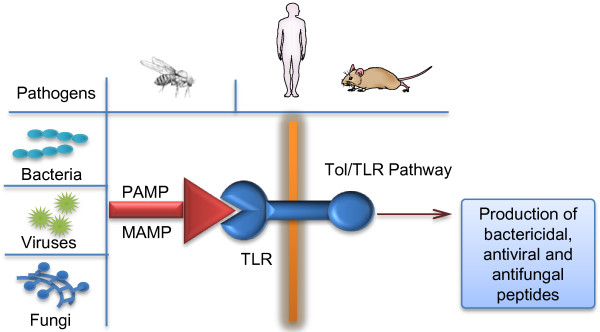
**Innate immune responses are elicited by ligation of specific cell surface receptors (for instance, TLR) recognizing molecular entities (PAMP and MAMP) shared by invaders, such as bacteria, viruses and fungi, but absent from the host.** PAMP: pathogen-associated molecular patterns; MAMP: microbe-associated molecular patterns; TLR: toll-like receptor.

**Figure 2:**
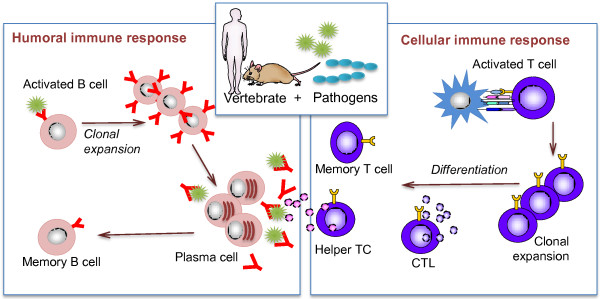
**Immune response in vertebrates to pathogens.** TC: T cells; CTL: cytotoxic T lymphocytes.

The vertebrate immune system evolved to maintain homeostatic balance between host tissues and the internal and external microbiological environment in order to assure the integrity of the host and possibly the microbiome (ie. the gut microbiota). In all multi-cellular organisms, both vertebrate and invertebrate, soluble factors are produced which protect against bacterial, viral and fungal invasion. In addition, in most animals, specialized cells developed which are dedicated to rapid recognition and elimination of pathogens by means of specific cell surface receptors (Figure 1) recognizing molecular entities such as pathogen-associated molecular patterns (PAMPs) shared by invaders but absent from the host. This system is referred to as innate, non-clonotypic immunity. Only in vertebrates (Figure 2), another class of cells emerged which recognize microorganisms by means of unique clonotypic receptors formed by recombination of highly diverse genetic modules to generate a very large repertoire of different antigen-recognizing molecules. On contact with their targets, these cells must undergo extensive clonal expansion and differentiation into effector cells in order to generate sufficient numbers of cells to successfully combat the invader. Thereafter, excess effector cells must be eliminated in a controlled manner by apoptosis, but a fraction of greatly varying size must be retained as memory cells to mediate a more rapid specific response on re-exposure to the same pathogen. Hence this arm of immunity is designated “adaptive”. Clearly, as the space available in the body is finite, there must be a limit to the amount of memory cells-vs-naïve cells that can be usefully maintained without overt leukocytosis. This is the theory of the restricted “immunological space” contributing to age-associated changes to immunity [1]. In most vertebrates, the receptors of the adaptive arm of immunity belong to the immunoglobulin family, expressed on the two main types of lymphocytes, B cells and T cells. Fascinatingly, in the few known surviving jawless vertebrates, the same division of labour is observed, and recently even a thymus candidate in lampreys has been discovered [2], but both the cell types and the genes for their clonotypic receptors are completely different (reviewed in [3]). This presumably represents an interesting case of evolutionary convergence and underscores the basic importance to all vertebrates of this dual type of adaptive immunity. Nonetheless, it remains a puzzle as to why invertebrates, which can be large and complex and often share the same external environment as vertebrates, seem able to protect themselves perfectly well against pathogenic microorganisms using innate immunity, whereas all vertebrates make what amounts to a considerable investment of resources in maintaining complex and potentially dangerous adaptive immune systems, using two completely different “tool kits”. An intriguing emerging theory to explain this posits that vertebrates have co-opted a much larger range of gut bacteria than invertebrates commonly have, in order to increase efficacy of nutrient processing. As innate immunity recognizes both beneficial and pathogenic microorganisms equally well via microbe-specific pattern receptors, it can be hypothesized that adaptive immunity arose to help distinguish between the two by regulating innate immunity and targeting specific antigens derived from pathogens but not from symbionts [4]. This implies that adaptive immunity must be able to deal with a much larger range of specificities than innate immunity, and hence the issue of the “immunological space” is of greater relative importance to the former.

Here, we will briefly review some of what is known about age-related changes to innate immunity in experimental models and in humans, and to adaptive immunity in higher animals, most particularly humans. It may be instructive to consider examples of invertebrates, which possess solely humoural innate defences (selecting C. elegans as the best, albeit still very superficially, investigated), then invertebrates with both cellular and humoural innate immunity (mostly Drosophila) and finally vertebrates possessing both innate and adaptive immunity (focussing on humans, except where reference must be made to another well-studied species: the mouse).

## Invertebrates

### C. elegans

**Figure 3:**
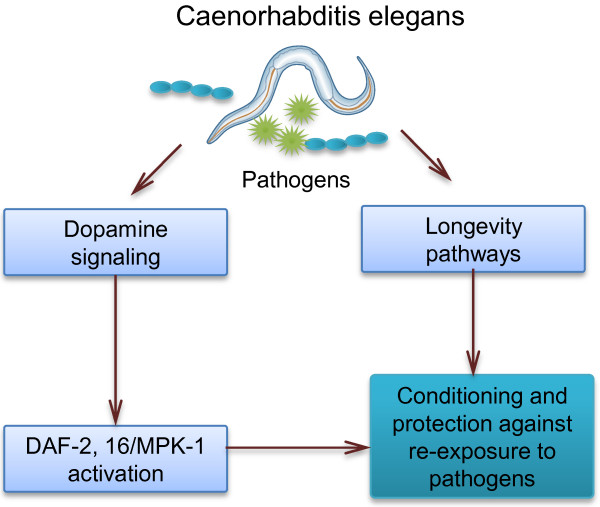
**Immune signalling pathways in C. elegans are linked with ageing.** DAF-2: insulin/insulin growth factor-1-like receptor; DAF-16: forkhead transcriptional factor; MAPK-1: mitogen-activated protein kinase 1.

Many basic lessons can be learned from the worm (Figure 3). First and foremost, strikingly, innate immune signalling pathways in C. elegans are intimately linked with ageing. In particular, the DAF-2 pathway is crucial for resistance to bacterial infection at the same time as being central to longevity determination [5-8]. Thus, older worms show increasing susceptibility to infection, and longer-lived DAF mutants are more resistant. In this model, it is possible to study purely humoural innate immunity, as no cell-mediated defences have been identified, reducing complexity of the analysis. The phylogenetically-widespread Toll-like receptors commonly employed by other invertebrates as well as mammals to recognize microbial products are also absent in worms, as is the conserved NF-κB transcription factor family, central to immune function in higher animals. Thus, C. elegans offers a simplified system for studying some aspects of immunity, without the confounding factors present in other models. Nonetheless, recent findings suggest that even C. elegans may possess some elements of immune memory, implying a central requirement for sophisticated immunity for survival even within such (relatively) simple systems. Thus, worms surviving exposure to a pathogen are able to survive re-exposure to doses of that pathogen which would otherwise have proven fatal. This ability is a central feature of adaptive immunity in higher organisms. How is this possible in the worm? It seems that the association between neurological and immune memory may already have emerged at the evolutionary stage of nematode development (Figure 3). Anyanful et al. found that this type of “immune memory” depended on the integrity of dopaminergic neurons, as well as signalling pathways crucial for longevity, such as DAF-2 and the p38 MAPK-like stress-resistance pathway regulating innate immunity [9]. Nonetheless, recent data show that ageing and innate immunity are regulated by distinct genetic mechanisms, confirming overlap but not identity in their genetic control [10]. The message from the worm, nonetheless, is clearly that immunity and aging are closely linked, not only via prevention of death due to infections [7,11].

### D. melanogaster

**Figure 4:**
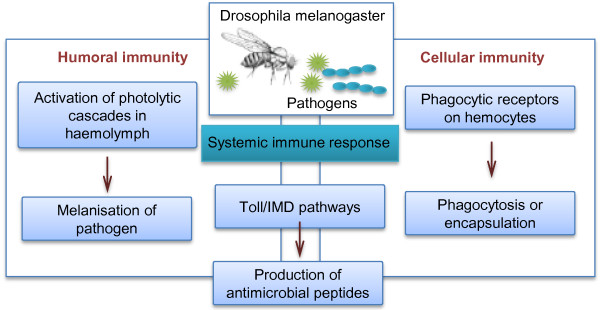
Systemic immune response in Drosophila melanogaster.

In addition to humoural immunity, like other insects as well as numerous other invertebrates, D. melanogaster (Figure 4) possesses cellular effectors expressing microbial pattern recognition receptors [12]. There are two main signalling pathways, designated Toll and IMD, both converging on NF-κB family transcription factors, which drive transcription of genes encoding anti-microbial peptides. The NF-κB family as a central regulatory complex is conserved throughout the higher metazoans; thus, unlike the innate pathways operative in C. elegans discussed above, these pathways are highly conserved and are also found in vertebrates. Similarly, a class of phagocytic cells analogous to mammalian macrophages is also present in insects (Figure 4) [13].

Despite the relative wealth of knowledge on insect immunity, surprisingly little is known about age-related changes to immune competence. Levels of anti-microbial peptides increase with age, and this has been associated with a decrease in fecundity [14] but not lifespan [15]. However, linking ageing with oxidative stress, gene transcription studies suggested that similar patterns of gene expression changes were induced in young flies exposed to oxidative stress as in old flies not otherwise stressed [16]; many of the genes upregulated with age were related to immune function [17]. More recently, infection models have suggested that old flies have more trouble clearing bacterial infections due to altered expression of genes involved in energy metabolism and insulin signalling [18]. One mechanism for this may be the decreased phagocytic activity of haemocytes in older flies [19]. However, there appears to be a great deal of heterogeneity between different populations of Drosophila, with age affecting immunity negatively but also positively in some lines [20]. Thus, much remains to be learned in this relatively unexplored area of comparative immunogerontology.

## Vertebrates

Vertebrates are unique in that in addition to retention of innate immunity of the types found in invertebrates, they also possess a sophisticated cellular and humoural immune system (Figure 2) exploiting clonotypic antigen receptors and imbued with memory (so-called “adaptive immunity”). They also possess other specialized cells that are part of the innate system but act as a bridge to the adaptive system and are not present in invertebrates. Thus, these cells (especially the antigen-presenting cells, DC, or NK cells) could also be responsible for age-associated decreased immunity in vertebrates; however, while changes are recorded, they do not seem as extravagant as those recorded for T and B cells (see following sections). Most information that we have on the ageing immune system in vertebrates derives from experimental studies in mice, with limited data also available in other rodents, cats, dogs, horses and non-human primates, in general consistent with the data from mice; extensive data are also available from observational studies in humans. Here, we will focus on the latter, but refer to rodent studies where human data are lacking or insufficient. Because, despite many similarities, mouse and human immunity can be different, we will indicate when referring to mouse data instead of human.

### Age associated differences in innate immunity in vertebrates

#### Phagocytic cells

**Figure 5:**
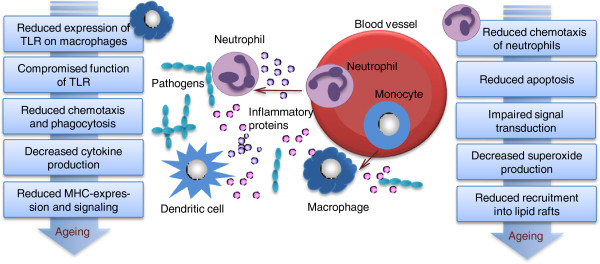
**Age-related changes to the “first-on-the-scene defenders”, such as macrophages and neutrophils.** TLR: toll-like receptor; MHC: major histocompatibility complex.

The first line of immune defence against microbial invasion (Figure 5), apart from physical barrier functions which may also degenerate with age but are not really part of immunity as such, is phagocytosis of pathogens by neutrophils, macrophages and other cells of this type, as in invertebrates. Thus, more extensive investigations of invertebrate models, where confounding factors include feedback from adaptive immune cells, should be valuable in researching this under-investigated facet of immunosenescence. These cells are attracted by factors released from damaged cells, both host and microbial components. As well as phagocytic activity, with destruction of the invaders by digestion, these first-on-the-scene defenders trigger inflammatory reactions by releasing soluble factors with systemic as well as local effects. It seems that the number and phagocytic capacity of the most numerous of these, the neutrophils, is relatively well-preserved in elderly people, but certain essential functions may be compromised [21]. The expression and function of essential surface receptors recognising microbe-specific molecules, such as Toll-like receptors, as well as cytokine production, and expression of other surface molecules like MHC class II antigens is also reduced [22].

#### Natural killer cells

**Figure 6:**
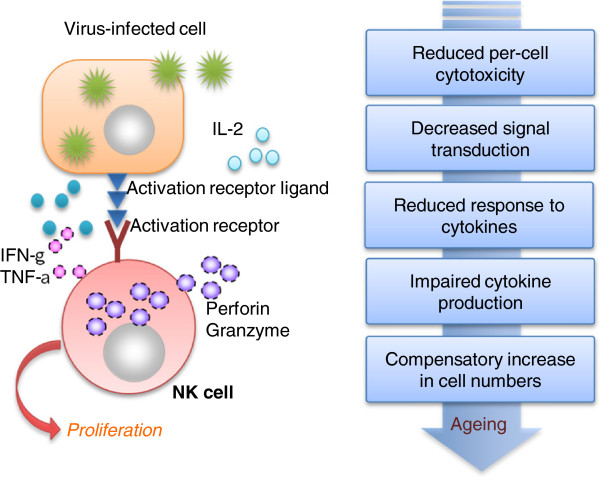
**NK cells and their age-related alterations.** NK cell: natural killer cell; I-2: Interleukin 2; IFN-γ: interferon gamma; TNF-α: tumor necrosis factor.

NK cells (Figure 6) represent a separate lineage of mononuclear lymphocytes which recognise and lyse virus-infected cells and tumours in an MHC-unrestricted manner and do not form memory, the hallmark of the adaptive immune response (although recently some memory-like functions have been ascribed to NK cells under certain circumstances). Rather little is known about alterations of NK activity in human ageing, with discrepant reports on numbers and functions of these cells. A consensus view could be proposed, that function on a per-cell basis decreases but the overall number of NK cells increases as a compensatory mechanism to maintain an important level of functionality. The relevance of NK cell activity for health and longevity is unclear, but a (lamentably small) number of studies does suggest that decreased NK cell function is associated with an increased incidence of infectious diseases and death due to infection in elderly adults [23]. NK function is probably important also for achieving a good response to influenza vaccination. Consistent with the importance of retaining NK function, it seems to be well-preserved in centenarians.

#### Dendritic cells

**Figure 7:**
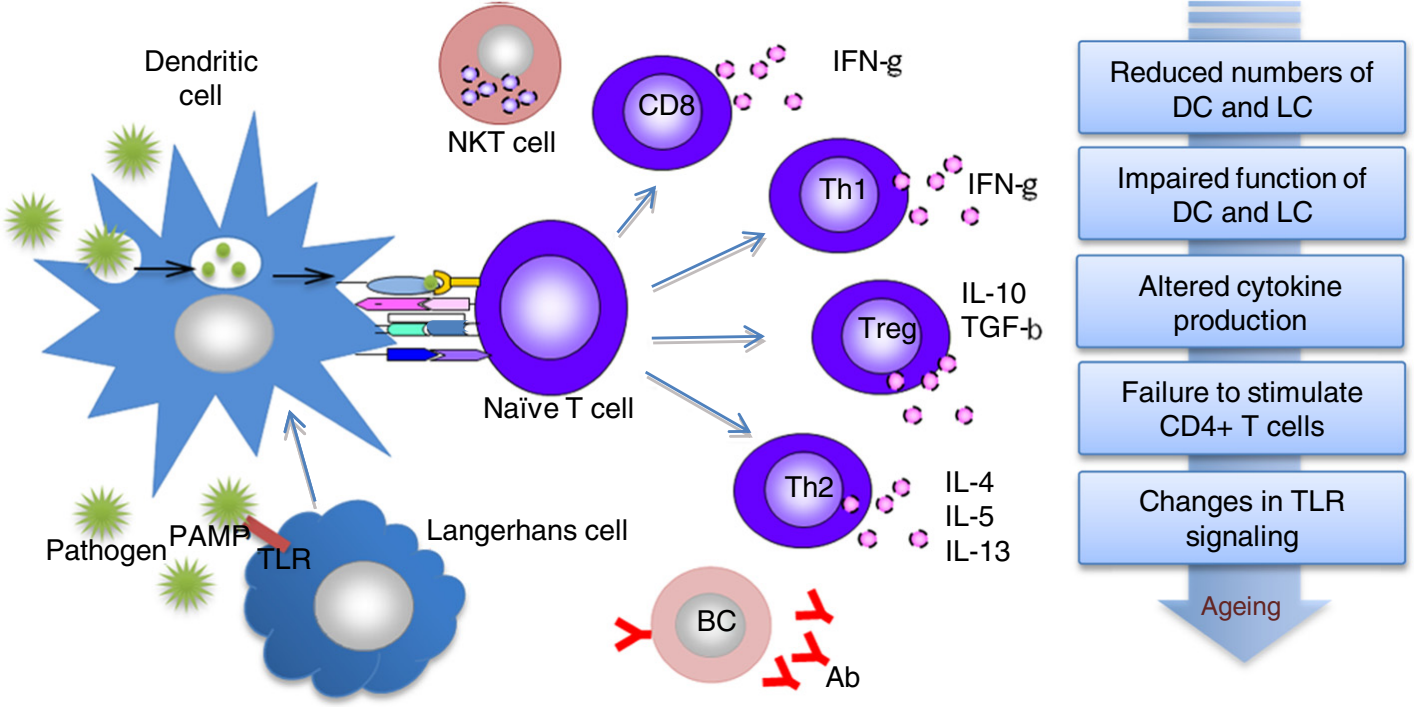
**Role of dendritic cells and Langerhans cells in immunity and their age-associated changes.** NKT cells: natural killer T cells; TLR: toll-like receptor; PAMP: pathogen-associated molecular pattern; BC: B cell; Ab: antibody; CD8: CD8-positive T cell; Th1: T helper 1 cell; Treg: regulatory T cell; Th2: T helper 2 cell; IFN-γ: interferon gamma; IL-10: interleukin 10; TGF-β: transforming growth factor beta; IL-4: interleukin 4: IL-5: interleukin 5; IL-13: interleukin 13.

Dendritic cells (DCs) form an essential bridge between the innate and adaptive immune systems, being responsible for recognising microbial-associated molecules, taking up and processing pathogen antigens, and presenting these to T cells (Figure 7). Without the proper function of DCs, no adaptive immune responses can take place; moreover, the manner in which the DCs present antigen controls the type of T cell response achieved, and thus the quality and intensity of adaptive immune responses. Peripheral blood is the most accessible source of DCs for direct analysis or to obtain monocyte-derived DCs. The numbers of DCs in the elderly may be reduced, and those present have more mature phenotypes and functions than in the young, consistent with their having already encountered and been stimulated by antigen. The numbers of skin DCs, termed Langerhans cells, are reduced in the elderly, and they seem to be functionally impaired to some extent. Nonetheless, most studies have concluded that antigen uptake, processing and presentation pathways in these innate immune cells appear to be relatively intact apart from some rather subtle differences. These include findings that DCs from young and elderly people stimulate naïve CD8^+^ T cells equally well, but those from the elderly may fail to stimulate naïve CD4^+^ T cells properly [24]. Some of these changes may be due to altered proportions of different types of APC [25], perhaps sufficing to explain the shifts in cytokine production by DC in the elderly that have been reported [26]. These would influence the nature of the naïve T cell response triggered by such DC; altered cytokine production by DCs and changes in TLR signalling may be attributable to altered signal transduction pathways involving phosphoinositide 3-kinase signalling [27] and, in mice, with reduced phosphorylation of STAT1 and STAT3 [28]. Failure to induce T cell responses would of course have grave consequences. For example, it has been shown in a mouse model that age-associated impairment of DC function led to defective T cell-mediated anti-tumor immunity [29]. Thus, our knowledge of age-associated changes to DCs is limited, but the rather subtle differences reported could have far-reaching “knock-on” effects, given that DCs are the central conductors at the innate-adaptive interface and that T cells, and therefore also B cells, are dependent for their function on these innate immune cells.

### Age-associated differences in adaptive immunity

**Figure 8:**
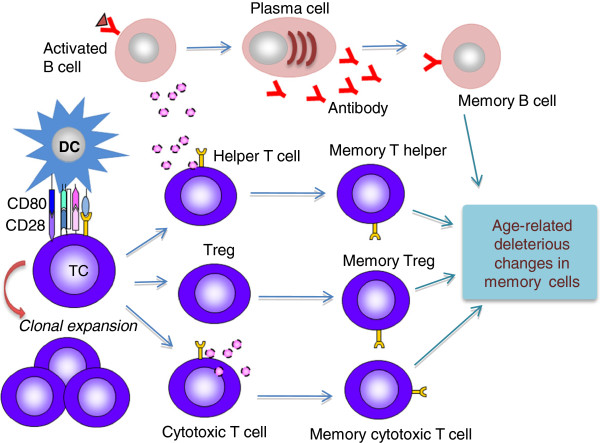
**Interaction of T cell and dendritic cell leading to T-cell activation, clonal expansion and differentiation.** If any of the activation requirements are detrimentally affected by ageing, immune responses will be compromised. Additionally, age-related deleterious changes in memory cells are to be expected. DC: dendritic cells; TC: T cells; Treg: regulatory T cells; CD: cluster of differentiation.

The adaptive immune response is orchestrated by T cell interactions with DCs, as alluded to above. T cells carrying specific receptors for the antigen fragments presented by DCs must not only be present, they must be able to respond properly (Figure 8). If the antigen derives from a pathogen not previously encountered, then naïve cells must be appropriately activated by antigen and costimulatory ligands on the DCs (eg. CD80, CD86) for which the T cells must also possess receptors (eg. CD28). These T cells must then undergo clonal expansion to generate sufficient cells from the limited number of precursors to mediate effective immune responses, ie. lysis of infected autologous cells, help for B cells to make antibody, cytokine and chemokine secretion to regulate tissue responses, and regulatory elements within the adaptive immune system to maintain control of ongoing responses. Thus, if any of these requirements are detrimentally affected by ageing, immune responses will be compromised. Reactivation requirements for re-exposure of memory cells at a later time point (sometimes decades later) to the same pathogens are less stringent, requiring less costimulation and less clonal expansion. Hence they are faster and more effective than primary responses, and possibly less susceptible to age-associated detrimental effects. However, if the primary response had occurred a long time ago (for most humans in the wild, this would have been in childhood), memory cells may be required to maintain themselves for decades in a host in the absence of antigen; thus, age-associated deleterious changes in these cells might also be expected. Clearly, there are many loci at which age-associated changes could kick in to alter immune function in the elderly. We will begin by considering the earliest phase of adaptive immunity, ie. the generation of naïve cells, which are plentiful in youth but may become “used up” following pathogen exposure and conversion to memory cells over the life course.

#### Thymic involution

**Figure 9:**
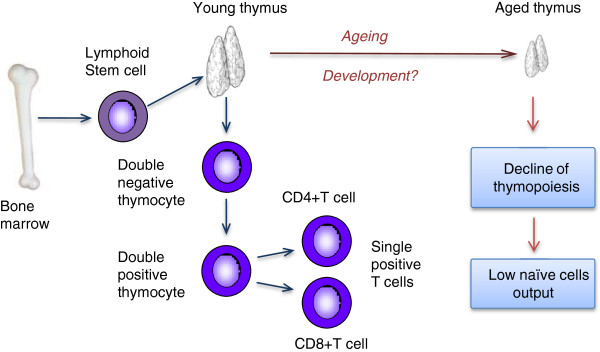
Thymic involution and its consequences for thymopoiesis.

**Figure 10:**
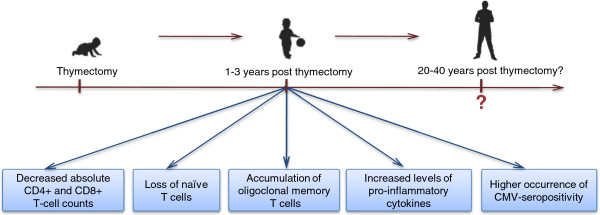
**Premature ageing of the immune system and signs of the immune risk profile in thymectomised patients.** CMV: cytomegalovirus.

Age-associated changes at the level of the hematopoietic stem cell (HSC) and bone marrow (BM) output reviewed elsewhere [30] suggest that it is the T and B cell compartments of adaptive immunity that are particularly affected, with innate immune components less so (with the proviso that the more marked changes in adaptive immunity may be at least in part the result of an amplification of the more subtle and more difficult to detect changes in the latter). Considering the clinical implications, regardless of whether interventions could be devised, or would then be desirable, to modulate HSC and/or the stem cell niche and restore youthful patterns of leukocyte output from the bone marrow, with less myeloid and more T and B lymphoid cells, larger numbers of T cell progenitors produced in this way would not be of any use unless the thymus retained functionality, and thymic selection processes were intact. This is because progenitors exported from the BM must undergo stringent processing in the thymus to generate mature functional naïve T cells (Figure 9). However, for reasons that remain essentially unclear, age-associated regression or “involution” of the thymus takes place at an early age, well before any chronological ageing occurs, ie. as part of developmental programming, not aging. This is true for all animals studied so far and thus strongly evolutionarily conserved (including birds, fish and amphibians as well as mammals) and is often said to be a central cause of T cell ageing, or immunosenescence. However, a process beginning during development cannot be considered as part of an ageing process; rather, reasons for thymic involution must be sought in developmental terms, and therefore attempts to intervene to prevent or reverse a ubiquitous developmental process must be viewed with extreme caution. Hypotheses to explain thymic involution remain unproven and have variously invoked energetic benefits [31], protection against leukemia [32], autoimmunity [33] and fine tuning the central positive T cell selection mechanism in the periphery [34]. Regardless of the reason, the outcome is that the capacity to produce functional naïve T cells decreases precipitously with age, starting even before puberty – but, on the other hand, even very elderly people commonly retain some small degree of residual thymic function [35]. This low-level output is perhaps sufficient to meet the needs of the old host, requiring only a small “top-up” of cells already present in the periphery, and still capable of clonal expansion. It could also be that the nature of the T cells generated is different in the elderly, as the thymus not only produces naïve T cells which develop into effector cells, but also regulatory T cells the targets of which are not pathogens, but as the name suggests, other T cells and components of the autologous immune system. Measuring T cell reconstitution after HSC transplantation (usually for cancer, mostly leukemia) into a depleted host mostly indicates very little retention of thymic activity in people aged 40 years or more [36]; but this situation in cancer patients may not reflect the normal state. Because recent thymic emigrants (RTEs) are naïve T cells which have not yet encountered antigen, the presumption has been that possessing fewer of them must be bad for the host, on the assumption that the individual’s capacity to resist infection with pathogens previously unencountered will be compromised. Thus, the elderly are thought to increasingly rely on naïve T cells produced earlier in life and possibly still present if not stimulated by their cognate antigen, together with the small numbers still being produced in most older people. Hence, the question can be justifiably asked whether the elderly do in fact experience clinical problems as a result of having low levels of naive T cells. Although this has been assumed, and because anecdotally the elderly suffer more from infections with emerging pathogens, such as SARS and West Nile Virus, there are very few systematic data available to support this contention. One datum supporting the idea that residual thymic output may indeed be clinically relevant is implied by a study of mortality in glioma patients, in which differences in the numbers of recent thymic emigrants seemed to explain the effect of age on outcome, but again, the subjects were cancer patients [37]. In this context, neonatal thymectomy (Figure 10) for cardiac surgery and the consequent decrease in numbers of naïve cells as well as other changes seen later in life [38] would be expected to have negative health consequences too, as has been argued, but again, there is little hard evidence for this so far. This may be because there is a general dearth of evidence, and because those children thymectomized early in life are not yet old enough to suffer the consequences. In any event, longitudinal studies have shown there appears to be little impact on infections and health at 1 and 3 years post-thymectomy [39,40], but this may be much too short a time to see any deleterious consequences. The situation is further complicated by the regrowth of the thymus in people theymectomized in infancy, whereby the deficit of naïve cells is minimized [41]. Thus, it remains the case that we have insufficient data from infant thymectomy to extrapolate to a conclusion that the decreased levels of naïve T cells often seen in elderly people can definitively be shown to be responsible for any deleterious clinical events. It is likely that the belief that vaccines are less effective in the elderly than in the young is true [42] but most of these are not novel agents stimulating naïve cells, but rather “booster” vaccines to agents already encountered or likely to cross-react with these. This is especially true for the most problematic of these, influenza, the poor response to which poses a serious public health problem in the elderly. The same may be said for shingles, tetanus and so on. These data therefore do not prove that fewer naïve cells in the periphery have a deleterious impact on the health of the elderly because much of the response would be expected to derive from memory cells. These considerations, of course, also raise the question as to whether the efforts of many investigators to “rejuvenate” thymic function, with its attendant dangers, might not be misguided and unnecessary. The above considerations are likely to apply to very elderly, mostly sessile individuals. However, with increasing activity and mobility of seniors, problems may emerge in terms of poor responses to travel vaccines, which would be expected to require the presence of naïve T cells and functional B cells to provide protection. Studies in this area are urgently required.

#### Antigen presentation

For the development of functional immunity, pathogens must be detected and responded to appropriately. For this purpose, surface receptors must bind specific ligand and trigger cell activation only when challenged with pathogen-derived molecules. As discussed above, the cells of the innate immune system achieve this by means of germline-encoded receptors recognizing a limited variety of targets present on micro-organisms but not found in the host (Figure 1), whereas cells of the adaptive immune system employ highly polymorphic receptors created by the random genetic recombination of diverse elements to assemble recognition units of enormous diversity. Antigens derived from pathogens must be taken up and processed by APC (most usually DCs as mentioned above) and presented on the cell surface to T cells (Figures 2, 7 and 8). It is therefore of paramount importance that both partners in this interaction are functioning in an optimal manner, both the T cells derived from potentially age-compromized HSCs and processed through a markedly age-affected thymus before being released to the periphery (Figure 9).

#### Immune cell function

**Figure 11:**
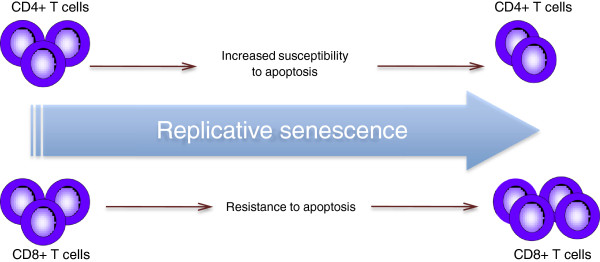
Accumulation of CD8-positive T cells and loss of CD4-positive T cells might be due to increased susceptibility or to acquired resistance to apoptosis of the respective T-cell subsets.

**T cell function at the population level** Experiments measuring T cell function in the elderly have commonly revealed marked differences compared with the young: less proliferative capability and decreased IL 2 production being repeatedly reported over the years [43]. These findings are commonly interpreted as evidence for decreased immune activity in the elderly, but may in fact merely reflect the altered proportions of naïve and memory cells in young and old, ie. a balance of the state of differentiation of the cell types present, as a result of previous exposures, or “immunological history”. In other words, these changes are adaptive. Naïve cells undergo vigorous clonal expansion involving IL 2 production; late-stage differentiated memory cells do not. In any experiments aiming to assess T cell status and function, failure to take this into account will merely result in a readout of differences in T cell distribution and functionality at different stages of differentiation from naïve to activated and memory cell, and the concurrent divergence of these cells along Th1, Th2 and other pathways (defined by commitment to selective expression of certain transcription factors, such as Tbet for Th1 cells, and the resulting cytokine secretion patterns, such as IFN-γ in the case of Th1 cells). This is of course important to know, but to compare young and old responses in the context of age-associated differences, it is necessary to compare like with like. Differences in the expression of surface receptors involved in lymphocyte homing, costimulation, adhesion etc (but not the antigen-recognising TCR) and in transcription factors determining T cell lineages, are commonly employed to distinguish “subsets” of T cells (although they are not actually fixed subsets in the way that CD4+ T cells and CD8+ T cells are, but in reality represent different dynamic stages of differentiation). According to one widely-employed model, naïve T cells express homing receptor CC-chemokine receptor 7 (CCR7) and leukocyte common antigen phosphatase isoform CD45RA, and have long telomeres, whereas many memory T cells express the alternative splice variant CD45RO, do not express CCR7 and have shorter telomeres, suggesting an extensive proliferative history (with lack of telomere maintenance). Isolating populations of T cells based on these markers assists in comparing like with like in the ageing context, but there is still the basic problem that we are not dealing with cell lineages here, but with differentiation stages. These will differ subtly in different individuals, and represents one of the many limitations to the most common type of study in humans, the cross-sectional study (where cohorts of different ages are compared with one another). This also makes it difficult to unequivocally identify “senescent” cells and to discern reliable biomarkers of immune ageing. However, the best accepted marker in this respect remains the relative levels of naïve T cells versus memory cells (which are heterogeneous, with a wide range of phenotypes and which also exhibit age-related differences in TCR diversity, telomere lengths and other parameters). This would be fine except that it is also very difficult to establish unequivocal markers by which to identify naïve T cells (and the proviso that we do not know for sure what clinical relevance low numbers of naïve cells actually has under most conditions of daily living either in elderly populations in industrialized countries or elsewhere). At the T cell population level, analysing the diversity of the TCR repertoire may be the most reliable guide to the naïve reserve of the individual. To mount an adequate response, a broad TCR repertoire must be maintained by ensuring the continuing presence of a diverse population of T-cell clones in preparation for exposure to completely unanticipated new pathogens. Molecular typing of the TCR gene repertoire, however, indicates that youthful diversity decreases with age, even within a particular group of T cells defined by surface markers as being at the same differentiation stage (“subset”). At least for CD4^+^ T cells, this has been reported to occur quite suddenly, with TCR diversity well maintained up to the age range of 60–65 years, despite the marked decrease of thymic output. However, repertoire diversity in 75–80 year olds is severely reduced and is suggested to contribute to poorer defence against infection in this age group [44]. However, this latter possibility has not actually been demonstrated and thus also remains a likely hypothesis still requiring testing. More data of this type are urgently required in different populations and with larger numbers of subjects. One explanation for these types of changes could be that because T cells are maintained in a state of constant turnover in the body, which is either antigen driven or homeostatic (see section below on lymphocyte dynamics), replicative senescence (proliferative exhaustion, of the type extremely well-studied in other cells, especially fibroblasts) can indeed occur. In cell culture, CD4^+^ T cells become more susceptible to apoptosis after extensive numbers of cell divisions [45,46] (Figure 11). The implication of these results is that by analogy *in vivo*, CD8^+^ T cells would accumulate and CD4^+^ T cells would be lost, and the CD4^+^:CD8^+^ T-cell ratio would therefore change. There is indeed evidence for the accumulation of CD8^+^ T cells *in vivo*, and for their correlation with poor outcomes to vaccination and with mortality, as discussed in more detail below. Nonetheless, the CD4:8 ratio commonly increases, rather than decreases, in most elderly people; the relatively rare exceptions to this may yield very important information on the immune status of the individual, putting them into the “immune risk profile” category [47].

**Figure 12:**
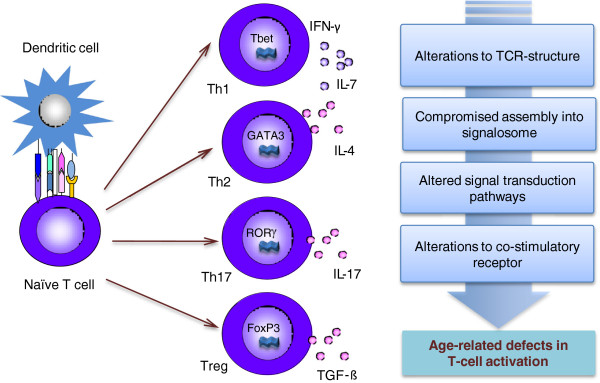
**T-cell differentiation into functionally different T-cell subsets and age-related defects in T-cell activation.** Th1: T helper 1 cells; Th2: T helper 2 cells; Th17: T helper 17 cells; Tbet, GATA3, ROR, FoxP3: different transcriptional factors; IL: interleukin; TGF-β: transforming growth factor beta.

**T-cell function at the single-cell level** As described above, to mediate a de novo adaptive immune response, naïve T cells must be activated by APCs and undergo extensive clonal expansion to generate sufficient effector cells to cope with the pathogen, followed by clonal contraction and development of memory at resolution of infection. Naïve T cells must therefore divide extensively, while differentiating into the appropriate balance of effector cells and memory cells to be retained at resolution of disease. The selection of functional differentiation pathways from naïve cell to numerous functional T cell populations is determined at this stage (Figure 12). These include an ever-growing family, designated Th1 (characterized as expressing the transcription factor Tbet, resulting in a certain pattern of cytokine gene transcription, including the hallmark pro-inflammatory cytokine IFN-γ), Th2 (expressing GATA3 and producing IL-4), Th17 (expressing RORγ and producing IL-17), regulatory CD4+ T cells (Tregs, expressing FoxP3 and producing TGF-β), etc. Alterations in numbers and functional integrity of T cell-surface receptors, both antigen-specific, and costimulatory, as well as their location in and transit through the membrane, and their intracellular signalling pathways, would all be expected to contribute to the final outcome of each T-cell–APC interaction over the large number of cell divisions required for a successful immune response. Studies have focused on alterations in signal transduction as well the formation of signalling domains (such as the immunological synapse) in a search for mechanisms responsible for any observed age-related defects in T cell activation. Alterations to the structure of the TCR or to co-stimulatory receptors do not appear obvious with advancing age, but their assembly into functional units (“signalosomes”) is probably compromised. Cell membrane fluidity has a major impact on TCR mobility and signalosome assembly, as originally suggested in early studies. The composition of the membrane changes according to systemic fatty-acid availability, not necessarily as a result of ageing per se. This is an example of an important confounding factor which is difficult to take into account in aging studies in humans, namely the impact of nutritional variation. Elderly people certainly differ from the young in their intakes of essentially all nutrients, often being deficient in multiple important components, but over-replete in others. The marked effect of the fatty acid environment was dramatically illustrated by a seminal study infusing lipids into young volunteers and measuring rapid alterations to T cell membrane lipid composition accompanied by depressed T cell functionality [48]. Increased levels of cholesterol also commonly seen in the elderly could contribute to age-associated deficits in T-cell signalling as well.

The number and relative proportions of cell surface receptors mediating co-stimulation are variable according to differentiation stage of the T cell such that they also change with age, disease and functional status. Indeed, decreased or absent expression of one of the well-studied costimulatory receptors, CD28, was proposed many years ago as a biomarker for immunosenescence [49]. This is consistent with findings that late-differentiated CD8+CD28^–^ T cells tend to accumulate particularly in the elderly in the immune risk profile group, and because CD28 expression is lost on *in vitro* culture prior to CD8 T cell replicative senescence. However, regulation of CD28 expression is likely to be part of the T cell differentiation program, rather than necessarily a sign of senescence. Moreover, CD28 may be up- or downregulated by cytokines such as IL-12 and TNF, respectively, independently of senescence**.** Altered signal transduction events in T cells have been much studied, but it remains unclear to what extent they are a result of altered proportions of the expressed positive and negative co-stimulatory receptors, like CD28 and its related negative signalling receptor CD152 (CTLA-4), or whether altered signal transduction pathways for each receptor, or both, are most important in determining the final outcome (ie. activation, anergy or apoptosis). In vitro studies on clonal cultured T cell populations may allow us to distinguish between changes caused by altered proportions of cell subsets and changes within the same clones at different times, eg. in the expression of the receptors alluded to above. Even for TCR signalling itself, differences between young and old donors´ naïve and memory T cells, or between CD4^+^ and CD8^+^ T cells, are not yet sufficiently well delineated.

**B cell function** In addition to the commonly decreased numbers of peripheral B cells in the elderly, especially those in the IRP group, age-associated changes to B cells and their subsets are also observed. These are less-well studied than in T cells, but like T cells, memory B cells do most likely accumulate with age, although data from different studies are discordant [50]. One reason for discrepancies might reside in the application of the markers identifying memory cells; as with T cells, it is differentiation states and not really subsets that are being measured [51]. At the functional level, age-associated alterations in immunoglobulin generation during immune responses have been reported, both via Ig class switching and somatic hypermutation [52]. This could be one mechanism accounting for the decline of the quality of humoral responses in the elderly. Further data on B cell alterations are needed, particularly since decreased absolute numbers of B cells may be associated with earlier mortality in longitudinal studies of the very elderly.

**Immune cell turnover** As discussed above, thymic output of naïve T cells is greatly decreased in older people, and often assumed to be negligible; nonetheless, in healthy elderly donors, peripheral T cell homeostasis is generally well maintained so that numbers of circulating T cells remain constant. How can this be accomplished? The answer is not likely to be extra-thymic generation of naïve T cells, but rather, the peripheral expansion of pre-existing T cells. Experiments to assess T cell dynamics are difficult in humans, but one pioneering approach exploits the incorporation of deuterated glucose into dividing lymphocytes (T cells, B cells and NK cells) to track their turnover. In naïve, memory and regulatory T-cells, glucose uptake and its subsequent loss occurs rapidly. Interestingly, it is the regulatory T cells that appear to proliferate the most rapidly [53]. One major important age-associated difference is that CD8^+^ memory cells in the elderly cycle considerably more slowly than any other subset, but this is not the case in young donors. This finding is consistent with the noted accumulation of CD8 memory cells in old people, since they may not reach a state of replicative senescence so quickly and are apoptosis-resistant [54], whereas the opposite is true of CD4+ T cells. B cells also showed some differences, but in this case it was the memory B cells that turned over more rapidly than the naïve cells. For NK cells, production and proliferation rates were lower in the elderly than in the young, which may also be relevant to the finding mentioned above that NK status is important for healthy ageing and infection resistance [23].

Because of the rapid T-cell turnover revealed by these studies**,** and given the Hayflick limit to the number of cell divisions normal somatic cells can undertake before senescence, it would be predicted that the proliferative capacity of the individual T cell clones constituting the response would eventually become exhausted [55]. Especially CD8 cells at this late stage express a highly differentiated phenotype (CD27 and CD28-negative, short telomeres, lack of telomerase and expression of negative signalling receptors like PD-1, CD57 and KLRG-1) [56]. In analogy with so-called replicatively senescent fibroblasts with altered cytokine secretion patterns, late-stage memory T-cells may also acquire new functions such as suppressive activity as previously demonstrated *in vitro* a decade earlier [57]. The mechanisms by which such “exhausted” cells suppress may include competition for cytokines or antigen on the DC surface, altered cytokine secretion patterns, or acquisition of cytotoxicity. At this stage, chronic stimulation by the original antigen itself is probably no longer necessary to keep the cells constantly turning over; hence memory T cells can persist for a lifetime, long after antigen exposure, even in the absence of persistent infection or periodic re-infection. Turnover may be supported by cytokines released by other cells reacting to completely different antigens (the so-called “bystander” effect). Thus, T-cell clonal exhaustion could be said to be an intrinsic characteristic of adaptive immunity, continuing even in the absence of specific chronic infection. However, in natural human populations, and even in sanitized Western societies, certain chronic infections or some other infections are always likely to be present. In every country studied so far, commonly persistent “asymptomatic” viruses and other pathogens which co-exist usually in a peaceful (perhaps even mutually beneficial?) association with the host are found. One of these, the beta-herpesvirus HHV5 (Cytomegalovirus, CMV), stands out from the rest as a common driving force for the observed age-associated immune alterations. Of course, this does not exclude the possibility that in different environments where CMV is less prevalent (should there be any, since CMV appears endemic in humans, and not being infected with it may actually be an artefact of civilisation), or for other reasons, other pathogens may play a similar role. Some similar phenomena linked to T cell clonal exhaustion, senescence and suppression may also occur in many disease states, such as in HIV infection, cancer and autoimmunity, in all of which different sources of antigen could act as a chronic immune stimulant. Thus, we would argue that age itself may not influence peripheral senescence events directly, but acts indirectly via the impact of differential pathogen loads and different durations of infection to exhaust the mature immune system [58]. In the absence of large-scale production of naïve T cells as a consequence of thymic involution and the limited capacity of the peripheral “immunological space” to absorb the accumulating memory cells, adaptive immunity becomes progressively compromised.

## Conclusions

Studying immunity in model organisms can be informative for determining the effects of age and might point the way to seeking previously unsuspected age-associated alterations in certain components of the human immune system otherwise obscured by the complexity of the whole. However, this is manifestly true only for components of innate immunity. The greater susceptibility to immunosenescence of the adaptive arm of immunity relative to the innate arm is suggested to be due to the necessity of maintaining clonal expansions of memory cells unable to self-renew in the way that innate immune cells can. This is the price paid for a much larger receptor repertoire of adaptive compared to innate immune cells, which in turn may be required to maintain homeostasis of the gut microbiota, keeping pathogens at bay while not reacting to the majority of symbionts.

## Competing interest

The authors declare no conflict of interest.

## Authors’ contributions

All authors read, approved and contributed to the writing of the final manuscript.
